# Expression of PPARγ and Paraoxonase 2 Correlated with *Pseudomonas aeruginosa* Infection in Cystic Fibrosis

**DOI:** 10.1371/journal.pone.0042241

**Published:** 2012-07-31

**Authors:** Phoebe E. Griffin, Louise F. Roddam, Yvonne C. Belessis, Roxanne Strachan, Sean Beggs, Adam Jaffe, Margaret A. Cooley

**Affiliations:** 1 Menzies Research Institute, Hobart, Tasmania, Australia; 2 School of Medicine, University of Tasmania, Hobart, Tasmania, Australia; 3 Department of Respiratory Medicine, Sydney Children’s Hospital, Randwick, New South Wales, Australia; 4 Department of Pediatrics, Royal Hobart Hospital, Hobart, Tasmania, Australia; The Scripps Research Institute, United States of America

## Abstract

The *Pseudomonas aeruginosa* quorum sensing signal molecule *N*-3-oxododecanoyl-l-homoserine lactone (3OC_12_HSL) can inhibit function of the mammalian anti-inflammatory transcription factor peroxisome proliferator activated receptor (PPAR)γ, and can be degraded by human paraoxonase (PON)2. Because 3OC_12_HSL is detected in lungs of cystic fibrosis (CF) patients infected with *P. aeruginosa,* we investigated the relationship between *P. aeruginosa* infection and gene expression of PPARγ and PON2 in bronchoalveolar lavage fluid (BALF) of children with CF. Total RNA was extracted from cell pellets of BALF from 43 children aged 6 months–5 years and analyzed by reverse transcription–quantitative real time PCR for gene expression of PPARγ, PON2, and *P. aeruginosa lasI,* the 3OC_12_HSL synthase. Patients with culture-confirmed *P. aeruginosa* infection had significantly lower gene expression of PPARγ and PON2 than patients without *P. aeruginosa* infection. All samples that were culture-positive for *P. aeruginosa* were also positive for *lasI* expression. There was no significant difference in PPARγ or PON2 expression between patients without culture-detectable infection and those with non-Pseudomonal bacterial infection, so reduced expression was specifically associated with *P. aeruginosa* infection. Expression of both PPARγ and PON2 was inversely correlated with neutrophil counts in BALF, but showed no correlation with other variables evaluated. Thus, lower PPARγ and PON2 gene expression in the BALF of children with CF is associated specifically with *P. aeruginosa* infection and neutrophilia. We cannot differentiate whether this is a cause or the effect of *P. aeruginosa* infection, but propose that the level of expression of these genes may be a marker for susceptibility to early acquisition of *P. aeruginosa* in children with CF.

## Introduction

Individuals with cystic fibrosis (CF) are particularly susceptible to infection with the opportunistic pathogen *Pseudomonas aeruginosa*, and ultimately over 80% of adults with CF patients are infected with this organism [1]. It is widely accepted that *P. aeruginosa* present in the lungs of CF patients, particularly during chronic infection, exist in the form of biofilms which protect the bacteria from both antibiotics and the host’s immune defenses. The reasons why CF patients are predisposed to *P. aeruginosa* infection are not clear, but it is known that infection is associated with more rapid decline in lung function [2], [3].

A hallmark of CF is a hyperinflammatory response to infection [4], [5], particularly with *P. aeruginosa* [6], [7], [8], [9]. The mammalian transcription factor, peroxisome proliferator activated receptor (PPAR)γ is a master negative regulator of inflammation, modulating signaling through NFκB and MAP kinases. It is expressed in respiratory epithelium [10] and has been reported to be expressed in immune cells such as macrophages and neutrophils [11], [12], [13], [14]. The expression and function of PPARγ have been reported to be low in human CF respiratory epithelial cell lines [10] and in cystic fibrosis transmembrane conductance regulator (*cftr*) knockout mice [15], [16]. This deficiency may contribute to the hyperinflammatory response in CF. To date this has not been confirmed in *ex vivo* samples from CF patients. However, PPARγ agonists have been reported to ameliorate intestinal symptoms in *cftr* knockout mice, and their potential use as therapy in chronic inflammatory disease has been widely discussed [17].

Biofilm formation and maturation of *P. aeruginosa*, together with the expression of a range of virulence factors, is regulated by quorum sensing signals that coordinate bacterial gene expression on a population-wide basis [18], [19]. These signal molecules have consistently been detected in nanomolar amounts in sputum and lung tissue from CF patients infected with *P. aeruginosa* [20] but localised concentrations are suggested to be in the micromolar range [21]. It has also been demonstrated that one of the major quorum sensing signal molecules of *P.aeruginosa*, *N*-3-oxododecanoyl-l-homoserine lactone (3OC_12_HSL), can cross the mammalian cell membrane [22] and that it induces hyperinflammatory responses in CF airway epithelial cell lines [6]. We [23] and others [24] have demonstrated that 3OC_12_HSL can bind to and modulate the function of PPARγ. We hypothesized that inhibition by 3OC_12_HSL of the function of an already low level of PPARγ expressed in CF could further exacerbate hyperinflammation in cystic fibrosis. To assess the viability of this hypothesis and to evaluate the level of PPARγ expressed in the lungs of children with CF, we examined PPARγ gene expression in cells from bronchoalveolar lavage fluid (BALF), comparing children with CF with and without demonstrated *P. aeruginosa* infection.

Another factor that could affect the efficiency of *P. aeruginosa* biofilm formation and virulence factor expression in the lung is the presence of mammalian lactonases, in particular paraoxonase 2 (PON2). PON2 is an intracellular enzyme that has been demonstrated to efficiently degrade 3OC_12_HSL [25], a process that can have a quorum quenching effect. Mouse tracheal epithelial cells deficient in PON2 have impaired ability to inactivate 3OC_12_HSL [26], and PON2 expression has been reported to be inhibited by 3OC_12_HSL [27]. We therefore also investigated the gene expression of PON2 in the cells from BALF of the children with CF.

We found that the expression of PPARγ and PON2 in BALF cells was significantly lower in patients infected with *P. aeruginosa* and was inversely correlated with total neutrophils in the BALF. Our results suggest that low PPARγ and PON2 expression is specifically associated with *P. aeruginosa* infection and neutrophilia in BALF.

## Materials and Methods

### Ethics Statement

Children with CF aged up to 5 years undergoing surveillance BAL between January 2009 and April 2011 and whose parents had given informed written consent for participation in a study of early lung disease at The Sydney Children’s Hospital, Randwick, Australia, contributed an aliquot of BALF for the current analyses. This study was approved by the South Eastern Sydney Area Health Service Human Research Ethics Committee (Approval no. 02/098) and registered at the Australian and New Zealand Clinical Trial Register (ACTRN12611000945921).

### Patients

Children with CF had been identified through newborn screening or meconium ileus presentation and the diagnosis confirmed by sweat chloride analysis (chloride concentration >60 mmol/mL) and/or *cftr* mutation analysis. Demographic and clinical variables were obtained from the patient’s medical records and are summarized in Table 1.

**Table 1:** Characteristics of all participants (43 patients).

Mean age at BAL(range)	2.25 years(6 months–5 years)
Male	19 (44.2%)
Homozygous F508del	21 (48.8%)
Azithromycin therapy at BAL	1 (2.3%)
Mean fasting glucose mmol/L(range)	4.83 (2.7–7.2)
*P. aeruginosa* infection* ^@^	7 (16.3%)
*S. aureus* infection* #	9 (20.9%)
*H. influenzae* infection* %	13 (30.2%)
Other bacteria infection*	6 (14.0%)
*A. fumigatus* infection* ∼	9 (20.9%)
No pathogen detected*	13 (30.2%)

* All pathogens detected by culture. Infection defined as >10^5^ cfu/mL BALF.

@Three of these samples also had S. aureus, one had both S. aureus and H. influenzae; one also had A. fumigatus.

# Four of these samples also had *P. aeruginosa* and four had *H. influenzae.*

% Three of these samples also had *S. aureus*; one had *P. aeruginosa.*

∼ One of these samples also had *P. aeruginosa.*

### BAL and Sample Processing

Flexible bronchoscopy with BAL was performed as previously described [28]. In brief, BAL was performed under general anesthesia. Suctioning through the bronchoscope was avoided until the tip had passed beyond the carina to avoid upper airway contamination. BAL was sequentially performed in three lobes, right upper lobe, right middle lobe and lingual, using a single aliquot (1 mL/kg, minimum 10 mL, maximum 20 mL) of sterile saline to each. BAL fluid samples were pooled.

For gene expression analysis, the cells contained in 0.5 mL of BALF were pelleted by centrifugation and stored in 300 µl of RNAlater® (Ambion, Mulgrave VIC, Australia) at −20°C until required. For a small number of patients, two samples taken at different times were available. Only the earliest sample from these patients was included in the main analysis.

### Detection of Bacteria in BALF

The presence of bacteria and fungi in BALF were evaluated by standard microbiological culture methods at Sydney Children’s Hospital. Airway infection for all microbes was defined by Sydney Children’s Hospital as pathogen growth >10^5^ colony-forming units per mL (cfu/mL) of BALF [29], [30].

### RNA Extraction and cDNA Synthesis

Total RNA was extracted from BALF cells using TRIzol® reagent (Invitrogen, Mulgrave, VIC, Australia). RNAlater® was removed from samples, and cells resuspended in 750 µl of TRIzol® before being transferred to a 2 ml tube containing 1 ml of 0.1 mm zirconia/silica beads (Daintree Scientific, St Helens, TAS, Australia.). A further 650 µl of TRIzol® was added before cells were homogenized for 2 min on maximum speed using a Mini-Beadbeater (Daintree Scientific.). Beads and cellular debris were pelleted by centrifugation at 10 000×*g* for 10 min at 4°C. Approximately 1 ml of supernatant was removed to a fresh tube and total RNA extracted as per the TRIzol® protocol with the addition of 5 µg of glycogen (Invitrogen) to the upper phase to aid RNA precipitation. Isolated RNA was resuspended in 25 µl of DEPC-treated water (Ambion) and DNAse treated using a TURBO DNA-free kit™ (Ambion) according to the manufacturer’s instructions. The concentration and purity of recovered RNA was quantified using a NanoDrop™ 1000 spectrophotometer (Thermo Scientific, Scoresby, VIC, Australia).

Up to 200 ng of total RNA was included in a cDNA synthesis reaction using a Transcriptor First Strand cDNA Synthesis kit (Roche, Dee Why, NSW Australia) according to the manufacturer’s instructions. The reaction included both anchored oligo d(T)_18_ and random hexamer primers to ensure reverse transcription of both mammalian and bacterial transcripts. cDNA was stored at −20°C until required.

### Quantitative Polymerase Chain Reaction (qPCR) Detection of PPARγ, PON2, Pseudomonas 16S rRNA and *lasI* Gene Expression

qPCR was performed on the LightCycler® 480 II PCR system (Roche). Duplicate 10 µl PCR reactions were performed using the LightCycler® 480 SYBR Green I Mastermix (Roche) according to the manufacturer’s instructions. Each reaction contained 0.75 µl cDNA and a final primer concentration of 300 nM. PCR conditions were as follows: 95°C for 5 min, then 45 cycles of 95°C for 15 s, 60°C for 15 s and 72°C for 30 s. All products underwent melt curve analysis. Primer sequences for human gene expression analysis were as follows:

PPARγ forward 5′-AGCTGAACCACCCTGAGTCC-3′

PPARγ reverse 5′-TCATGTCTGTCTCCGTCTTCTTG-3′

PON2 forward 5′-GCCAACAATGGGTCTGTTCTCC-3′

PON2 reverse 5′-CAGCTTCCCATCATACACTGAGGC-3′

β-actin forward 5′-GGCTGGCCGGGACCTGACTGA-3′

β-actin reverse 5′-CTTCTCCTTAATGTCACGCACG-3′.

PPARγ primers detected transcripts for PPARγ isoforms 1 and 2. Primers used for the detection of *P. aeruginosa* 16S rRNA [31] and *lasI* [32] have been published elsewhere. Human gene expression relative to the housekeeping gene β-actin was calculated using 2^−ΔCp^. The presence of *P. aeruginosa* 16S rRNA in BAL fluid samples was defined as positive by both Cp and melt curve analysis, with level of infection defined as high (Cp<20), medium (Cp≥20 and <30) and low (Cp≥30 and ≤40). Negative for *P. aeruginosa* by PCR was defined as the absence of specific PCR product. Detection of *lasI* expression was defined as the presence of specific PCR product and Cp≤40 cycles.

### Statistical Analysis

All statistical analyses were performed using Graphpad Prism v4.03 (Graphpad Software, La Jolla, CA, USA). Microbiological culture results were coded positive or negative. Genotype was coded as F508del homozygous, F508del heterozygous or other. Results of RT–qPCR for *P. aeruginosa* were coded as negative, low, medium or high and *lasI* was coded as negative/positive. Age at BAL was expressed in days, and gene expression of PPARγ and PON2 calculated relative to that of the reference gene, β-actin. Because PPARγ and PON2 gene expression levels were not normally distributed, all analysis was performed using nonparametric statistics: Mann–Whitney *U* test for comparisons between two groups, Kruskal–Wallis analysis with Dunn’s multiple comparison test for comparisons between multiple groups and Spearman correlation for assessing correlations between continuous variables. Fisher’s exact test was used to evaluate associations between categorical variables.

## Results

### Gene Expression of PPARγ in BALF Cells of Children with CF is Lower in those Who have *P. aeruginosa* Infection

**Figure 1:**
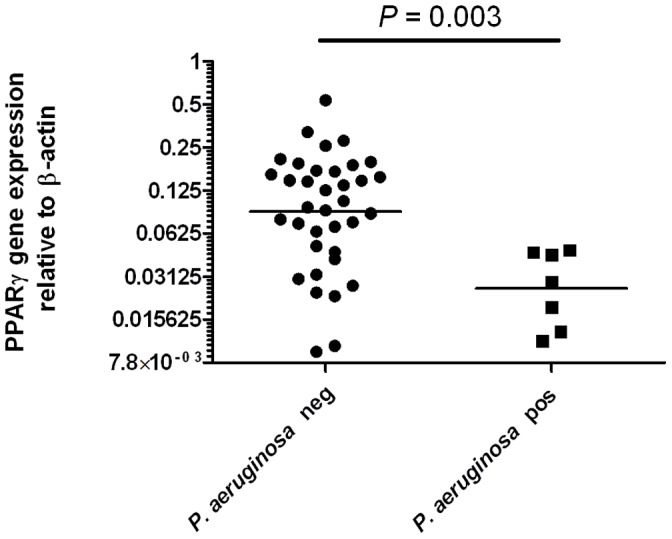
Relative gene expression of PPARγ in BALF from CF patients with and without culture-defined *P. aeruginosa* infection. Horizontal bars represent median values. Significance assessed by Mann–Whitney *U* test. *P. aeruginosa* pos  =  *P. aeruginosa* infection detected by culture (>10^5^ cfu/mL BALF). *P. aeruginosa* neg  =  *P. aeruginosa* undetectable by culture.

Cells from BALF samples from all subjects were examined for PPARγ gene expression by RT–qPCR, for the presence of *P. aeruginosa* by culture and RT–qPCR, and for the presence of any other pathogen by culture only. “Culture positive” and airway infection were defined as detection of >10^5^ cfu/mL in BALF [29], [30]. Figure 1 shows that PPARγ gene expression was significantly lower (approximately threefold, *P* = 0.003, Mann–Whitney *U* test) in BALF from children with *P. aeruginosa* infection than in those without *P. aeruginosa* infection.

We also assessed the presence and level of *P. aeruginosa* and the expression of *lasI* in BALF by RT–qPCR. Semi-quantitation of *P. aeruginosa* 16S RNA was performed based on Cp as described in the Materials and Methods; *lasI* expression was classified as positive or negative. All samples that were culture-positive for *P. aeruginosa* were also positive for *lasI* expression by RT–qPCR. All other samples were negative for *lasI*, including those that were culture negative but where low or medium levels of *P. aeruginosa* were detected by RT–qPCR. As shown in Table 2, there was an excellent correlation between detection of high or medium levels of *P. aeruginosa* 16S rRNA expression in BALF by RT–qPCR and culture detection of >10^5^ cfu/mL *P. aeruginosa* (*P*<0.0001, Fisher’s exact test), with RT–qPCR showing a sensitivity of 86% and specificity of 97% relative to culture.

**Table 2:** Comparison of culture and RT–qPCR for detection of *P. aeruginosa* and *lasI.*

	High or medium byRT–qPCR	Low or negative byRT–qPCR	*lasI* positive
Culture positive	6	1	7
Culture negative	1	35	0

**Figure 2:**
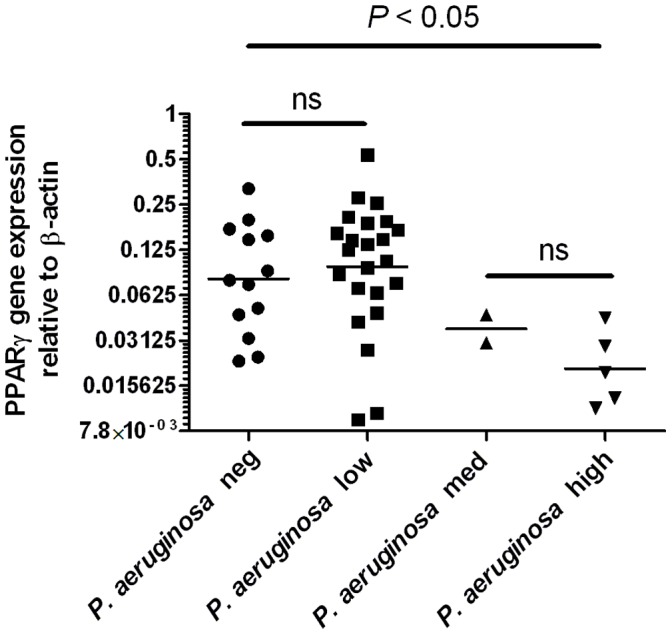
Relative gene expression of PPARγ in BALF from CF patients with *P. aeruginosa* detected by RT–qPCR. Horizontal bars represent median values. Significance assessed by Kruskal–Wallis analysis with Dunn’s post test. *P. aeruginosa neg*  =  no detectable 16S rRNA product in PCR. *P. aeruginosa* low  =  product detected Cp>30; *P. aeruginosa* med  =  product detected Cp 20–30; *P. aeruginosa* high  =  product detected Cp<20.

We also evaluated PPARγ gene expression in cells from BALF based on *P. aeruginosa* RT–qPCR results (Figure 2), which confirmed that detection of medium or high levels of *P*. *aeruginosa* was correlated with low gene expression of PPARγ.

### Gene Expression of PPARγ is Low in Children with *P. aeruginosa* Infection but not in those Infected with Other Pathogens

**Figure 3:**
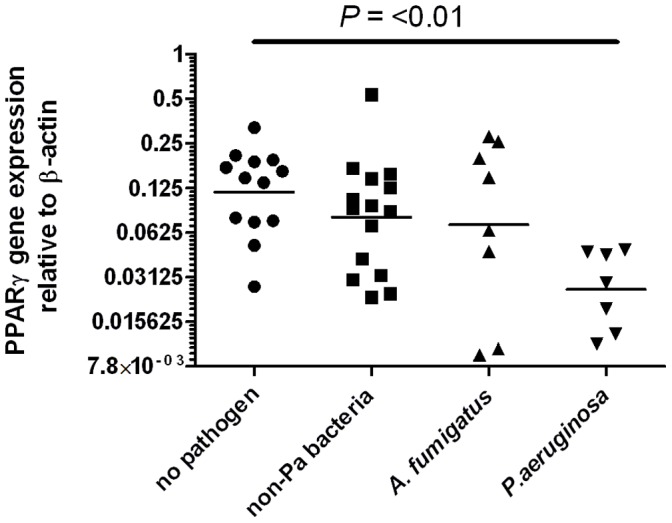
Low PPARγ gene expression is associated with *P. aeruginosa* infection but not with presence of other pathogens. Horizontal bars represent median values. Significance compared with no pathogen group assessed by Kruskal–Wallis analysis with Dunn’s post test. No pathogen: no bacterial or fungal pathogens detected by culture. non-Pa bacteria: infection with any bacteria other than *P. aeruginosa* without detectable *P. aeruginosa* and *A. fumigatus*; *A. fumigatus*: infection with *A. fumigatus* without detectable *P. aeruginosa* and with or without other bacteria; *P. aeruginosa*: infection with *P. aeruginosa* with or without other pathogens. Infection defined as >10^5^ cfu/mL BALF.

Most patients had more than one pathogen detected in BALF, so we were unable to meaningfully analyze the effects on PPARγ gene expression of infection with each individual type of microbe because of possible interactions between them. For example, there were three patients with only *P. aeruginosa* infection, three patients with both *P. aeruginosa* and *S. aureus*, one with *P. aeruginosa*, *S. aureus* and *H. influenza*, three with both *S. aureus* and *H. influenzae,* three with only *S. aureus* and eight with only *H. influenzae* infection. There were no patients with a combination of *P. aeruginosa* and *H. influenzae.* Therefore, to determine whether low PPARγ gene expression was merely the result of infection in general, or was specifically associated with *P. aeruginosa* infection, we used culture results to divide patient samples into four groups: those without detected pathogens (n = 13), those with *P. aeruginosa* alone or in combination with other pathogens (n = 7), those with *H. influenzae* and/or with *S. aureus* (non-Pseudomonal bacteria group, n = 15) and those with *Aspergillus fumigatus* with or without non-Pseudomonal bacteria (n = 8). The results shown in Figure 3 demonstrate that only the presence of *P. aeruginosa* was associated with significantly lower PPARγ gene expression.

### Gene Expression of PON2 is Low in Patients with *P. aeruginosa* Infection but not with other Bacterial Infections

**Figure 4:**
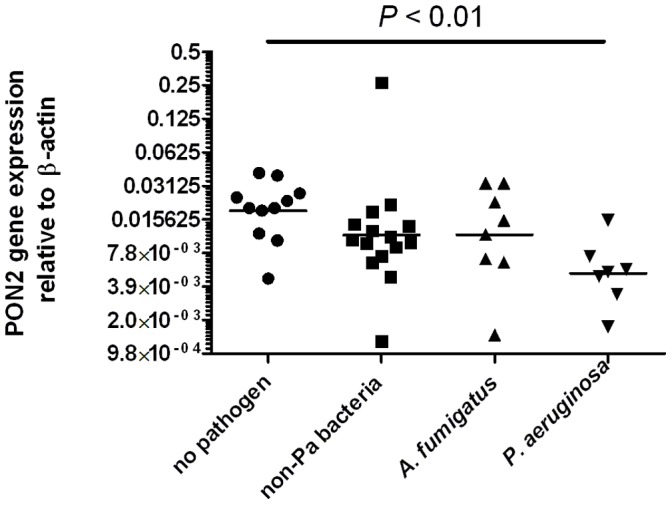
Low PON2 gene expression is associated with *P. aeruginosa* infection but not with other pathogens. Horizontal bars represent median values. Significance compared with no pathogen group assessed by Kruskal–Wallis analysis with Dunn’s post test. No pathogen: no bacterial or fungal pathogens detected by culture. non-Pa bacteria: infection with any bacteria other than *P. aeruginosa* with undetectable *P. aeruginosa* and A. fumigatus; *A. fumigatus*: *Aspergillus fumigatus* infection without detectable *P. aeruginosa* and with or without other bacteria; *P. aeruginosa*: *P. aeruginosa* infection with or without other pathogens. Infection defined as >10^5^ cfu/mL BALF.

Because the human lactonase PON2 has been shown to efficiently inactivate and degrade the *P. aeruginosa* signaling molecule 3OC_12_HSL that can modulate PPARγ activity, we were interested in evaluating whether the gene expression of PON2 was also correlated with *P. aeruginosa* infection. We hypothesized that if PON2 expression was also low in patients with *P. aeruginosa* infection, then this could contribute to their susceptibility to *P. aeruginosa* infection because they would have a reduced ability to degrade and inactivate 3OC_12_HSL. The results shown in Figure 4 confirm that PON2 gene expression is approximately twofold lower in those children with *P. aeruginosa* infection than in those infected with non-Pseudomonal bacteria or without detectable infection. There was one sample in the no infection group for which PON2 expression data was unavailable, leaving 12 patients in that group.

### Correlation of Other Variables with *P. aeruginosa* Infection and with PPARγ and PON2 Gene Expression

The results described above indicate an association between *P. aeruginosa* infection and low gene expression of PPARγ and PON2. To analyze this further, we evaluated associations of a range of variables with the presence of >10^5^ cfu/mL *P. aeruginosa* in BALF (“Pseudomonas positive”) (Table 3) or PPARγ and PON2 gene expression (Table 4). To evaluate the association of PPARγ and PON2 gene expression with inflammation, we used the total leukocyte and total neutrophil counts in BALF. As the severity of CF disease has been reported to differ between males and females [33], [34]and will also depend on the type of *cftr* mutation present in the patients, we evaluated associations with sex and genotype. The effect of patient age was also evaluated, because the incidence of *P. aeruginosa* in CF increases with age, and in some animal models gene expression of PPARγ has been reported to change with age [35], [36]. Lastly, because PPARγ function is important in regulating insulin sensitivity [37] and polymorphisms in PON2 have been associated with diabetes [38], we evaluated associations with fasting blood glucose levels. We were unable to obtain glucose tolerance test results for many patients, although this is a more reliable measure of CF-related diabetes [39]. We also considered the use of azithromycin, because this antibiotic has been reported to modulate quorum sensing and biofilm formation in *P. aeruginosa* [40], but as only one patient was being treated with azithromycin at the time of BAL, this variable was not included in the analysis. Neutrophil and total leukocyte counts were significantly higher in *P. aeruginosa*-positive samples (*P* = 0.006 and *P* = 0.009 respectively) compared with negative samples. The results showed no significant differences between *P. aeruginosa*-positive and *P. aeruginosa*-negative samples for any of the other variables.

**Table 3:** Comparison of characteristics of patients with and without culture-detected *P. aeruginosa* infection in BALF.

Variable	*P. aeruginosa* positive(n = 7)	*P. aeruginosa* negative(n = 36)	*P-*value
Age at BAL (days, mean ± SEM)	1072±204.3	760.9±72.5	0.23*
No. male (%)	3 (42.9%)	16 (44.4%)	0.50^#^
No. F508del homozygous (%)	2 (28.5%)	19 (52.8%)	0.24^#^
Total leukocytes in BALF (cells ×10^9^/L, mean ± SEM	**1.09±0.19**	**0.57±0.11**	**0.009** *
Total neutrophils in BALF (cells ×10^9^/L, mean ± SEM	**0.84±0.17**	**0.34±0.10**	**0.006** *
Fasting glucose mean ± SEM	4.94±0.21	4.81±0.14	0.66*

* Mann Whitney *U* test ^#^Chi square. Significant differences are indicated in bold text.

**Table 4:** Correlation of patient characteristics with PPARγ and PON2 gene expression.

Variable	Correlation with PPARγ gene expression (Spearman *r*, *P* value)	Correlation with PON2 gene expression (Spearman *r*, *P* value)
Age at BAL	0.013, *P* = 0.9326	−0.168, *P* = 0.2880
Sex	0.220, *P* = 0.1557	0.000, *P* = 1.000
Genotype	−0.012, *P* = 0.9390	−0.060, *P* = 0.7050
Total leukocytes in BALF	−**0.478, ** ***P*** ** = 0.0016** *	−0.297, *P* = 0. 0.062
Total neutrophils in BALF	−**0.635, ** ***P*** **<0.0001** *	−**0.376, ** ***P*** ** = 0.018** *
Fasting glucose	−0.042, *P* = 0.7982	0.314, *P* = 0.0545

* Significant correlations are indicated in bold text.

When evaluating associations between each variable and PPARγ expression, the only correlations observed were an inverse correlation between PPARγ expression and total leukocytes (*P* = 0.0016), and an extremely strong inverse correlation for PPARγ expression and total neutrophils (*P*<0.0001); i.e. low PPARγ gene expression is associated with high total leukocyte and neutrophil counts. For PON2, there was an inverse correlation of PON2 gene expression with total neutrophils (*P* = 0.018), but no other significant correlations.

### PPARγ and PON2 Gene Expression in Different BALF Samples from Individual Patients

To attempt to evaluate whether *P. aeruginosa* infection was associated with changes in PPARγ and PON2 gene expression in individual patients, we seperately examined two consecutive BALF samples from six patients who either acquired *P. aeruginosa* infection between the first and second sample (n = 3), or cleared *P. aeruginosa* infection between the first and second sample (n = 3). There was no consistent pattern of change in PPARγ or PON2 gene expression. Results from another group of 16 patients for whom we had at least two BALF samples but who did not change their *P. aeruginosa* infection status (either stayed negative or stayed positive) also showed no significant change in median PPARγ and PON2 gene expression between samples.

## Discussion

Previous studies in human cell lines and *cftr* knockout mice have suggested that expression of PPARγ is reduced in CF [10], [15], [16], although this has not been directly demonstrated in individuals with CF, and no previous study has suggested an association of PPARγ expression with *P. aeruginosa* infection. There is no information in the literature on the expression of PON2 directly in CF. Thus, this study is the first to examine expression of the genes encoding PPARγ and PON2 in cells from BALF of CF children with and without *P. aeruginosa* infection.

The results of this study show that in children with CF, low expression of PPARγ and PON2 genes in BALF cells is associated with *P. aeruginosa* infection but not with the presence of other pathogens. Patients with *P. aeruginosa* infection had significantly higher neutrophil and leukocyte counts compared to those without, which has been previously reported [41]. The findings also demonstrate that PPARγ gene expression, and to a lesser extent PON2 gene expression, shows a strong inverse correlation with neutrophil counts, indicating that low PPARγ and PON2 gene expression is associated with high levels of inflammation. It is possible that the observed correlation is due to sampling from different cell populations and we were unable to determine the source of detected PPARγ transcripts in our study. However PPARγ has been reported to be expressed in bronchial epithelial cells [10], [42], alveolar macrophages [14] and neutrophils [11], [12], [13].

These findings were consistent whether *P. aeruginosa* infection was detected by culture or by RT–qPCR. While microbiological culture remains the gold standard detection method, our results indicate that detection by RT–qPCR shows an excellent correlation with culture results. Furthermore, detection of low levels of *P. aeruginosa* in patients classified on culture results as uninfected suggests that RT–qPCR may detect lower levels of bacteria than culture, although the clinical relevance of this is uncertain. However, the findings suggest that RT–qPCR could be a useful confirmatory technique for *P. aeruginosa* infections.

Our results also showed that expression of the bacterial 3OC_12_HSL synthase *lasI* gene was detectable in BALF of all patients with culture-defined *P. aeruginosa* infection, but in none of the other patients. This confirms that at least a subpopulation of the *P. aeruginosa* present in BALF in all patients with culture-defined infection with *P. aeruginosa* were able to produce 3OC_12_HSL, even in those patients who had been infected for up to three years. This contrasts with reports that isolates of *P. aeruginosa* from chronically-infected CF patients accumulate mutations and lose quorum sensing activity [43], [44]. However, these studies reported quorum sensing gene mutations in clinical isolates rather than population-wide gene expression directly in the CF lung. It is likely that mixed populations of *P. aeruginosa* exist in the CF lung, some of which may harbor mutations in genes involved in quorum sensing [45], [46]. In addition, it has been reported that quorum sensing activity in isolates is not lost until the late stages of chronic infection [44]. It is important to emphasize that our study reports data on gene expression, not protein activity, of PPARγ and PON2. However, a positive regulatory loop where increased functional protein leads to increased gene expression, and inhibition of protein function leads to decreased mRNA expression, has been demonstrated for PPARγ [47] and other PPAR family members PPARα [48] and PPARδ [49]. Thus, there is good evidence that gene expression and protein function of PPARγ are closely correlated. Our finding that expression of the PPARγ gene is strongly inversely correlated with the presence of an inflammatory neutrophil infiltrate is also indirect evidence that PPARγ transcriptional activity is likely to be reduced in *P. aeruginosa*-positive patients, because PPARγ protein is a transcription factor that is known to downregulate inflammatory gene expression [37]. Although information about the association between PON2 gene expression and protein function is limited, it is known that substrate inhibition of gene expression and probably protein levels occurs [27]. Thus, while it remains necessary to confirm the levels of functional PPARγ and PON2 protein in these patients, evidence suggests that these too are likely to be reduced.

This was primarily a cross-sectional study and although we had paired BALF samples from five patients who acquired or cleared *P. aeruginosa* infection, changes in gene expression between these samples were inconsistent. The changes in gene expression between these patients did not differ from changes seen in paired samples from 10 patients who did not change *P. aeruginosa* infection status. Because of the limited number of samples, we were unable to conclusively show that expression of PPARγ and PON2 genes either changed or remained the same in individual patients upon infection with *P. aeruginosa*. Thus our data did not allow us to determine whether low PPARγ predisposes patients to early acquisition of *P. aeruginosa*. Our hypothesis that 3OC_12_HSL inhibits PPARγ function in CF lungs remains plausible, because we showed expression of *lasI,* the 3OC_12_HSL synthase, in the BALF of all patients with culture-defined *P. aeruginosa* infection. However, confirmation of this hypothesis requires further study, including analysis of expression of PPARγ protein levels and function, that is not possible with the limited material available from BALF. The alternative possibility, that low PPARγ expression predisposes CF patients to early acquisition of *P. aeruginosa*, also remains plausible, and could provide a useful screening test that might allow preemptive treatment in those children at higher risk. This requires further studies of sequential samples from individual patients, to determine whether those who have low PPARγ expression acquire *P. aeruginosa* earlier in life or at a higher frequency than patients with higher PPARγ expression.

Our observation that lower expression of the PON2 gene is associated with *P. aeruginosa* infection in CF patients is also of importance. PON2 is a member of the paraoxonase family of human enzymes that have lactonase activity [25] and can inactivate and degrade 3OC_12_HSL [25], [50], [51]. Thus, reduced PON2 activity could facilitate chronic infection and biofilm formation by *P. aeruginosa* by allowing higher levels of functional 3OC_12_HSL to accumulate in the CF lung. Indeed, PON2-deficient mouse tracheal epithelial cells allow increased *P. aeruginosa* quorum sensing activity [50] and PON2 expression has been shown to be downregulated by 3OC_12_HSL [27]. Further, PON2 has antioxidant activity and can protect cells against the effects of the *P. aeruginosa* virulence factor pyocyanin [27]. Importantly, PON2 has activity against a range of homoserine lactone signaling molecules, including that produced by *Burkholderia cepacia* complex, but as none of our patients had detectable infection with this microorganism, we were unable to evaluate any associations.

Overall, our study represents the first demonstration of two host factors that are specifically associated with early childhood infection with *P. aeruginosa* in individuals with CF. While our results do not allow us to determine whether this association is cause or effect, they provide a useful starting point for designing new therapeutic strategies. For example, treating CF patients who have low PPARγ expression with a pharmacological PPARγ agonist such as a glitazone may allow augmentation of PPARγ activity and provide some protection against *P. aeruginosa* infection.

## References

[1] Lyczak JB, Cannon CL, Pier GB (2002) Lung infections associated with cystic fibrosis. Clin Microbiol Rev 15: 194–222. 11932230 10.1128/CMR.15.2.194-222.2002 PMC118069

[2] Jarad NA, Higgs S, Jeffcote T, Giles K (2005) Factors associated with reduced FEV1 in adult patients with cystic fibrosis in a relatively affluent area. Chron Respir Dis 2: 133–137. 16281436 10.1191/1479972305cd065oa

[3] Schaedel C, de Monestrol I, Hjelte L, Johannesson M, Kornfalt R, et al (2002) Predictors of deterioration of lung function in cystic fibrosis. Pediatr Pulmonol 33: 483–491. 12001283 10.1002/ppul.10100

[4] Ulrich M, Worlitzsch D, Viglio S, Siegmann N, Iadarola P, et al (2010) Alveolar inflammation in cystic fibrosis. J Cyst Fibros 9: 217–227. 20347403 10.1016/j.jcf.2010.03.001 PMC2883667

[5] Machen TE (2006) Innate immune response in CF airway epithelia: hyperinflammatory? Am J Physiol Cell Physiol 291: C218–230. 16825601 10.1152/ajpcell.00605.2005

[6] Mayer ML, Sheridan JA, Blohmke CJ, Turvey SE, Hancock RE (2011) The Pseudomonas aeruginosa Autoinducer 3O-C12 Homoserine Lactone Provokes Hyperinflammatory Responses from Cystic Fibrosis Airway Epithelial Cells. PLoS One 6: e16246. 21305014 10.1371/journal.pone.0016246 PMC3031552

[7] Buchanan PJ, Ernst RK, Elborn JS, Schock B (2009) Role of CFTR, Pseudomonas aeruginosa and Toll-like receptors in cystic fibrosis lung inflammation. Biochem Soc Trans 37: 863–867. 19614608 10.1042/BST0370863

[8] Saadane A, Soltys J, Berger M (2006) Acute Pseudomonas challenge in cystic fibrosis mice causes prolonged nuclear factor-kappa B activation, cytokine secretion, and persistent lung inflammation. J Allergy Clin Immunol 117: 1163–1169. 16675347 10.1016/j.jaci.2006.01.052

[9] Smith RS, Kelly R, Iglewski BH, Phipps RP (2002) The Pseudomonas autoinducer N-(3-oxododecanoyl) homoserine lactone induces cyclooxygenase-2 and prostaglandin E2 production in human lung fibroblasts: implications for inflammation. J Immunol 169: 2636–2642. 12193735 10.4049/jimmunol.169.5.2636

[10] Perez A, van Heeckeren AM, Nichols D, Gupta S, Eastman JF, et al (2008) Peroxisome proliferator-activated receptor-gamma in cystic fibrosis lung epithelium. Am J Physiol Lung Cell Mol Physiol 295: L303–313. 18556801 10.1152/ajplung.90276.2008 PMC2519842

[11] Greene ME, Blumberg B, McBride OW, Yi HF, Kronquist K, et al (1995) Isolation of the human peroxisome proliferator activated receptor gamma cDNA: expression in hematopoietic cells and chromosomal mapping. Gene Expr 4: 281–299. 7787419 PMC6134382

[12] Reddy RC, Narala VR, Keshamouni VG, Milam JE, Newstead MW, et al (2008) Sepsis-induced inhibition of neutrophil chemotaxis is mediated by activation of peroxisome proliferator-activated receptor-{gamma}. Blood 112: 4250–4258. 18535203 10.1182/blood-2007-12-128967 PMC2582007

[13] Standiford TJ, Keshamouni VG, Reddy RC (2005) Peroxisome proliferator-activated receptor-{gamma} as a regulator of lung inflammation and repair. Proc Am Thorac Soc 2: 226–231. 16222042 10.1513/pats.200501-010AC

[14] Asada K, Sasaki S, Suda T, Chida K, Nakamura H (2004) Antiinflammatory roles of peroxisome proliferator-activated receptor gamma in human alveolar macrophages. Am J Respir Crit Care Med 169: 195–200. 14563653 10.1164/rccm.200207-740OC

[15] Andersson C, Zaman MM, Jones AB, Freedman SD (2008) Alterations in immune response and PPAR/LXR regulation in cystic fibrosis macrophages. J Cyst Fibros 7: 68–78. 17889625 10.1016/j.jcf.2007.05.004

[16] Harmon GS, Dumlao DS, Ng DT, Barrett KE, Dennis EA, et al (2010) Pharmacological correction of a defect in PPAR-gamma signaling ameliorates disease severity in Cftr-deficient mice. Nat Med 16: 313–318. 20154695 10.1038/nm.2101 PMC2834836

[17] Becker J, Delayre-Orthez C, Frossard N, Pons F (2006) Regulation of inflammation by PPARs: a future approach to treat lung inflammatory diseases? Fundam Clin Pharmacol 20: 429–447. 16968414 10.1111/j.1472-8206.2006.00425.x

[18] Miller MB, Bassler BL (2001) Quorum sensing in bacteria. Annu Rev Microbiol 55: 165–199. 11544353 10.1146/annurev.micro.55.1.165

[19] Smith RS, Iglewski BH (2003) P. aeruginosa quorum-sensing systems and virulence. Curr Opin Microbiol 6: 56–60. 12615220 10.1016/s1369-5274(03)00008-0

[20] Chambers CE, Visser MB, Schwab U, Sokol PA (2005) Identification of N-acylhomoserine lactones in mucopurulent respiratory secretions from cystic fibrosis patients. FEMS Microbiol Lett 244: 297–304. 15766782 10.1016/j.femsle.2005.01.055

[21] Charlton TS, de Nys R, Netting A, Kumar N, Hentzer M, et al (2000) A novel and sensitive method for the quantification of N-3-oxoacyl homoserine lactones using gas chromatography-mass spectrometry: application to a model bacterial biofilm. Environ Microbiol 2: 530–541. 11233161 10.1046/j.1462-2920.2000.00136.x

[22] Ritchie AJ, Whittall C, Lazenby JJ, Chhabra SR, Pritchard DI, et al (2007) The immunomodulatory Pseudomonas aeruginosa signalling molecule N-(3-oxododecanoyl)-L-homoserine lactone enters mammalian cells in an unregulated fashion. Immunol Cell Biol 85: 596–602. 17607318 10.1038/sj.icb.7100090

[23] Cooley MA, Whittall C, Rolph MS (2010) Pseudomonas signal molecule 3-oxo-C12-homoserine lactone interferes with binding of rosiglitazone to human PPARgamma. Microbes Infect 12: 231–237. 20074659 10.1016/j.micinf.2009.12.009

[24] Jahoor A, Patel R, Bryan A, Do C, Krier J, et al (2008) Peroxisome proliferator-activated receptors mediate host cell proinflammatory responses to Pseudomonas aeruginosa autoinducer. J Bacteriol 190: 4408–4415. 18178738 10.1128/JB.01444-07 PMC2446782

[25] Teiber JF, Horke S, Haines DC, Chowdhary PK, Xiao J, et al (2008) Dominant role of paraoxonases in inactivation of the Pseudomonas aeruginosa quorum-sensing signal N-(3-oxododecanoyl)-L-homoserine lactone. Infect Immun 76: 2512–2519. 18347034 10.1128/IAI.01606-07 PMC2423076

[26] Stoltz DA, Ozer EA, Ng CJ, Yu JM, Reddy ST, et al (2007) Paraoxonase-2 deficiency enhances Pseudomonas aeruginosa quorum sensing in murine tracheal epithelia. Am J Physiol Lung Cell Mol Physiol 292: L852–860. 17122353 10.1152/ajplung.00370.2006

[27] Horke S, Witte I, Altenhofer S, Wilgenbus P, Goldeck M, et al (2010) Paraoxonase 2 is down-regulated by the Pseudomonas aeruginosa quorumsensing signal N-(3-oxododecanoyl)-L-homoserine lactone and attenuates oxidative stress induced by pyocyanin. Biochem J 426: 73–83. 19925453 10.1042/BJ20091414

[28] Dakin CJ, Numa AH, Wang H, Morton JR, Vertzyas CC, et al (2002) Inflammation, infection, and pulmonary function in infants and young children with cystic fibrosis. Am J Respir Crit Care Med 165: 904–910. 11934712 10.1164/ajrccm.165.7.2010139

[29] Armstrong DS, Grimwood K, Carlin JB, Carzino R, Olinsky A, et al (1996) Bronchoalveolar lavage or oropharyngeal cultures to identify lower respiratory pathogens in infants with cystic fibrosis. Pediatr Pulmonol 21: 267–275. 8726151 10.1002/(SICI)1099-0496(199605)21:5<267::AID-PPUL1>3.0.CO;2-K

[30] Hall GL, Logie KM, Parsons F, Schulzke SM, Nolan G, et al (2011) Air trapping on chest CT is associated with worse ventilation distribution in infants with cystic fibrosis diagnosed following newborn screening. PLoS One 6: e23932. 21886842 10.1371/journal.pone.0023932 PMC3158781

[31] Matsuda K, Tsuji H, Asahara T, Kado Y, Nomoto K (2007) Sensitive quantitative detection of commensal bacteria by rRNA-targeted reverse transcription-PCR. Appl Environ Microbiol 73: 32–39. 17071791 10.1128/AEM.01224-06 PMC1797142

[32] Wargo MJ, Hogan DA (2007) Examination of Pseudomonas aeruginosa lasI regulation and 3-oxo-C12-homoserine lactone production using a heterologous Escherichia coli system. FEMS Microbiol Lett 273: 38–44. 17559399 10.1111/j.1574-6968.2007.00773.x

[33] Dodge JA, Lewis PA, Stanton M, Wilsher J (2007) Cystic fibrosis mortality and survival in the UK: 1947–2003. Eur Respir J 29: 522–526. 17182652 10.1183/09031936.00099506

[34] Jackson AD, Daly L, Jackson AL, Kelleher C, Marshall BC, et al (2011) Validation and use of a parametric model for projecting cystic fibrosis survivorship beyond observed data: a birth cohort analysis. Thorax 66: 674–679. 21653925 10.1136/thoraxjnl-2011-200038 PMC3142345

[35] Herzlich AA, Ding X, Shen D, Ross RJ, Tuo J, et al (2009) Peroxisome Proliferator-Activated Receptor Expression in Murine Models and Humans with Age-related Macular Degeneration. Open Biol J 2: 141–148. 21152244 10.2174/1874196700902010141 PMC2998287

[36] Hotta K, Bodkin NL, Gustafson TA, Yoshioka S, Ortmeyer HK, et al (1999) Age-related adipose tissue mRNA expression of ADD1/SREBP1, PPARgamma, lipoprotein lipase, and GLUT4 glucose transporter in rhesus monkeys. J Gerontol A Biol Sci Med Sci 54: B183–188. 10361996 10.1093/gerona/54.5.b183

[37] Pascual G, Sullivan AL, Ogawa S, Gamliel A, Perissi V, et al (2007) Anti-inflammatory and antidiabetic roles of PPARgamma. Novartis Found Symp 286: 183–196; discussion 196–203. 18269183 10.1002/9780470985571.ch16

[38] Mackness B, McElduff P, Mackness MI (2005) The paraoxonase-2–310 polymorphism is associated with the presence of microvascular complications in diabetes mellitus. J Intern Med 258: 363–368. 16164576 10.1111/j.1365-2796.2005.01554.x

[39] Hameed S, Jaffe A, Verge CF (2011) Cystic fibrosis related diabetes (CFRD)–the end stage of progressive insulin deficiency. Pediatr Pulmonol 46: 747–760. 21626717 10.1002/ppul.21495

[40] Tateda K, Comte R, Pechere JC, Kohler T, Yamaguchi K, et al (2001) Azithromycin inhibits quorum sensing in Pseudomonas aeruginosa. Antimicrob Agents Chemother 45: 1930–1933. 11353657 10.1128/AAC.45.6.1930-1933.2001 PMC90577

[41] Sagel SD, Gibson RL, Emerson J, McNamara S, Burns JL, et al (2009) Impact of Pseudomonas and Staphylococcus infection on inflammation and clinical status in young children with cystic fibrosis. J Pediatr 154: 183–188. 18822427 10.1016/j.jpeds.2008.08.001 PMC2654617

[42] Honda K, Marquillies P, Capron M, Dombrowicz D (2004) Peroxisome proliferator-activated receptor gamma is expressed in airways and inhibits features of airway remodeling in a mouse asthma model. J Allergy Clin Immunol 113: 882–888. 15131570 10.1016/j.jaci.2004.02.036

[43] Mowat E, Paterson S, Fothergill JL, Wright EA, Ledson MJ, et al (2011) Pseudomonas aeruginosa population diversity and turnover in cystic fibrosis chronic infections. Am J Respir Crit Care Med 183: 1674–1679. 21297072 10.1164/rccm.201009-1430OC

[44] Bjarnsholt T, Jensen PO, Jakobsen TH, Phipps R, Nielsen AK, et al (2010) Quorum sensing and virulence of Pseudomonas aeruginosa during lung infection of cystic fibrosis patients. PLoS One 5: e10115. 20404933 10.1371/journal.pone.0010115 PMC2853559

[45] Wilder CN, Allada G, Schuster M (2009) Instantaneous within-patient diversity of Pseudomonas aeruginosa quorum-sensing populations from cystic fibrosis lung infections. Infect Immun 77: 5631–5639. 19805523 10.1128/IAI.00755-09 PMC2786440

[46] Sandoz KM, Mitzimberg SM, Schuster M (2007) Social cheating in Pseudomonas aeruginosa quorum sensing. Proc Natl Acad Sci U S A 104: 15876–15881. 17898171 10.1073/pnas.0705653104 PMC2000394

[47] Takamura T, Nohara E, Nagai Y, Kobayashi K (2001) Stage-specific effects of a thiazolidinedione on proliferation, differentiation and PPARgamma mRNA expression in 3T3-L1 adipocytes. Eur J Pharmacol 422: 23–29. 11430909 10.1016/s0014-2999(01)01053-6

[48] Pineda Torra I, Jamshidi Y, Flavell DM, Fruchart JC, Staels B (2002) Characterization of the human PPARalpha promoter: identification of a functional nuclear receptor response element. Mol Endocrinol 16: 1013–1028. 11981036 10.1210/mend.16.5.0833

[49] Shureiqi I, Jiang W, Zuo X, Wu Y, Stimmel JB, et al (2003) The 15-lipoxygenase-1 product 13-S-hydroxyoctadecadienoic acid down-regulates PPAR-delta to induce apoptosis in colorectal cancer cells. Proc Natl Acad Sci U S A 100: 9968–9973. 12909723 10.1073/pnas.1631086100 PMC187904

[50] Stoltz DA, Ozer EA, Ng CJ, Yu J, Reddy ST, et al (2006) Paraoxonase-2 Deficiency Enhances Pseudomonas aeruginosa Quorum Sensing in Murine Tracheal Epithelia. Am J Physiol Lung Cell Mol Physiol. 10.1152/ajplung.00370.2006 17122353

[51] Ozer EA, Pezzulo A, Shih DM, Chun C, Furlong C, et al (2005) Human and murine paraoxonase 1 are host modulators of Pseudomonas aeruginosa quorum-sensing. FEMS Microbiol Lett 253: 29–37. 16260097 10.1016/j.femsle.2005.09.023

